# Neurite Damage in Patients with Migraine

**DOI:** 10.3390/neurolint16020021

**Published:** 2024-02-29

**Authors:** Yasushi Shibata, Sumire Ishiyama

**Affiliations:** 1 Department of Neurosurgery, Headache Clinic, Mito Medical Center, University of Tsukuba, Mito Kyodo General Hospital, Mito 3100015, Japan; 2 Center for Medical Sciences, Ibaraki Prefectural University of Health Sciences, Ami 3000394, Japan

**Keywords:** migraine, neurite, MRI

## Abstract

We examined neurite orientation dispersion and density imaging in patients with migraine. We found that patients with medication overuse headache exhibited lower orientation dispersion than those without. Moreover, orientation dispersion in the body of the corpus callosum was statistically negatively correlated with migraine attack frequencies. These findings indicate that neurite dispersion is damaged in patients with chronic migraine. Our study results indicate the orientation preference of neurite damage in migraine.

## 1. Introduction

Migraine has a high prevalence and causes high levels of disability worldwide and is ranked second among the diseases in terms of the years lived with disability in the Global Burden of Disease Study in 2016 [1]. The International Classification of Headache Disorders, third edition (ICHD-3), is the world’s standard classification and diagnostic criteria of headache [2]. This classification and diagnostic criteria for migraine are based only on a patient’s history, symptoms, and clinical findings. Calcitonin-gene-related peptide (CGRP) is the molecule responsible for the pathology of migraine [3]. Specific anti-CGRP monoclonal or anti-CGRP receptor antibodies have been developed and are clinically effective in preventing migraine attacks [4,5,6]. The small molecules of CGRP antagonists have also been used for both the prevention and acute treatment of migraine attacks in some countries [7,8,9,10]. The CGRP concentration in saliva is higher in chronic migraine (CM) patients than in episodic migraine (EM) patients [11,12]. However, the diagnostic credibility of CGRP concentration in saliva has not been investigated. Thus, to the best of our knowledge, no biomarker has been identified for diagnosing migraine.

Neuroimaging is a standard clinical examination used to diagnose neurological diseases. Magnetic resonance imaging (MRI) is noninvasive and has high spatial resolution and high contrast; thus, it is used to diagnose various brain diseases, including cerebrovascular diseases, brain tumors, traumatic hematomas, epilepsy, congenital neural diseases, hydrocephalus, intracranial infection, psychiatric diseases, and neurodegenerative diseases [13,14,15,16,17,18].

Patients with migraine have a higher prevalence of cerebellar and brain stem infarction and white matter T2 hyperintense lesions than healthy controls according to the Cerebral Abnormalities in Migraine, Epidemiological Risk Analysis study [19,20]. The authors of this study discussed the possibility of impaired adaptive cerebral hemodynamic mechanisms in the posterior circulation of migraine patients being the cause of these ischemic lesions [20]. Another study on women with migraine showed a strong trend toward an association with lacunar stroke and no association with atherosclerotic and cardio-embolic strokes [21]. This study indicated no difference in the risk of stroke between the anterior and posterior regions. The association between migraine and cerebral infarction and white matter hyperintense lesions has been reported [22,23,24]. Most of these studies have a cross-sectional design; thus, the causal and temporal associations of these regions with migraine are unclear. In one longitudinal study [22], the progression of white matter hyperintense lesions was not observed among migraine patients during the 8–12-year study period. They speculated that brain lesions in migraine patients may occur early in life.

Migraine is predominantly observed in young women [25,26]. Migraine with aura, oral hormonal contraceptives, and tobacco smoking are risk factors for ischemic stroke in young women [21,24,27,28,29]. Although the incidence of ischemic stroke among young women is low, women with migraine with aura should be advised to stop smoking or stop using oral contraceptives.

Patent foramen ovale causes intracardiac right–left shunt and paradoxical embolus. Cases of cryptogenic stroke accompanied by migraine have a high prevalence (79%) among patients with patent foramen ovale who have right-to-left shunting [30]. A meta-analysis of case–control studies revealed an association between the presence of patent foramen ovale and migraine with aura but not with migraine without aura [31]. Given that cerebral ischemia lesions are related to migraine with aura, microembolization and ischemia are speculated to be the cause of cortical spreading depression [32], which is the primary cause of migraine aura [33,34]. However, a series of studies of regional cerebral blood flow during migraine attacks showed reduced regional cerebral blood flow posteriorly spreading slowly and contiguously anteriorly and crossing borders of the supply of major cerebral arteries [35]. Human studies showed initial hyperemia followed by prolonged hypoperfusion. Therefore, cortical spreading depression is not simply caused by ischemia. Moreover, most migraine attacks are not accompanied by aura, which are then diagnosed as migraines without aura. Therefore, cortical spreading depression could not fully explain the pathology of most migraines [35].

Migraine premonitory symptoms, including general fatigue, irritability, food cravings, and yawning, were observed 2 days before the migraine attacks [36]. The activation of the hypothalamus demonstrated by functional MRI [37] is considered the pathology of these premonitory symptoms. Therefore, the hypothalamus is considered the generator of migraine attacks.

Cerebral microbleeds are indicators of cerebral vascular damage. Migraine patients showed a significantly higher prevalence of infratentorial microbleeds than healthy controls [38]. However, standard anatomical MRI revealed no abnormalities that are specific for patients with migraine.

Volume analysis, i.e., volumetry, is a popular method for analyzing brain morphological changes. Migraine patients showed decreased gray matter volume in the posterior–opercular regions, prefrontal cortex, and anterior cingulate cortex compared with healthy controls [39]. Most volume studies were cross-sectional and compared migraine patients with healthy controls. Liu et al. analyzed the longitudinal MRI volume changes in patients with migraine and reported progressive gray matter volume reductions in the dorsolateral and medical parts of the superior frontal gyrus, orbitofrontal cortex, hippocampus, precuneus, and primary and secondary somatosensory cortices during the 1-year observation period in patients with early migraine without aura [40]. In this study, changes in diffusion tensor imaging (DTI), which is a method used to measure the direction of water diffusion, were not observed during the same period [41].

Cerebral cortical thickness was investigated using MRI. Compared with healthy controls, patients with migraine showed cortical thickening in the caudal somatosensory cortex, which is responsible for the head and face [42]. Another study showed cortical thickening in the left rostral middle frontal and bilateral postcentral gyri [43]. The average thickness of the bilateral postcentral gyri was positively correlated with migraine disease duration and headache frequency. Another study indicated cortical thinning at the somatosensory cortex, right fusiform gyrus, and right temporal pole in patients with migraine, as compared with healthy controls [44]. These cortical thickness changes were investigated in relation to migraine attack frequencies. Somatosensory cortical thickness was thin and thick in low- and high-frequency episodic migraine (EM) patients, respectively, as compared with healthy controls [45]. Another study indicated reduced cortical thickness and cortical surface area in the regions subserving pain processing, and conversely, the cortical thickness and surface area were increased in the migraine in regions involved in executive functions and visual motion processing [46]. These cortical thickness and surface area abnormalities were related to aura and white matter hyperintensities but not to migraine disease duration and attack frequency. In this study, the cortical surface area abnormalities were more pronounced and more widely distributed than the cortical thickness abnormalities. Cortical surface area normally increases during late fetal development as a consequence of cortical folding, whereas cortical thickness changes throughout the life span as a consequence of development and disease. Therefore, these results might indicate a congenital condition rather than consequences of repeated migraine attacks. The discrepancies between several studies may be explained by different patient populations, analytical and statistical methods, applied thresholds, and included covariates. Neural plasticity must have significant effects, especially on cortical thickness, in the long term.

However, some abnormal findings of advanced MRI methods, including DTI and functional MRI (fMRI), have been reported [47,48,49,50,51]. The amount of longitudinal and perpendicular diffusions are expressed as axial diffusivity (AD) and radial diffusivity (RD), respectively, and the mean value of the three mutually perpendicular diffusivities is referred to as the mean diffusivity (MD) [41]. Fractional anisotropy (FA) is defined as the index of the directionality of diffusion. In free water, water molecules freely diffuse in every direction; hence, FA is defined as 0. If diffusion is restricted to only one direction, FA becomes 1. In the central nervous system, homogenously directed neural fibers, including the corpus callosum and internal capsule, have high FA values; hence, these regions are good targets for DTI investigation. We previously reported significantly lower FA in the white matter of patients with migraine with aura and medication overuse headache using 1.5-Tesla MRI [47]. Planchuelo-Gómez et al. reported DTI findings in migraine patients [52]. They found considerably reduced AD in CM patients as compared with EM patients. Nonetheless, these findings are insufficient for diagnosing migraine at present. Therefore, a new biomarker for migraine diagnosis is required.

Neurite orientation dispersion and density imaging (NODDI) is an advanced imaging method that automatically divides tissues into the following three components: free water, extraneurite, and intraneurite [53]. The fraction of isovolume (Fiso), which is the fraction of free water, is a marker of inflammation [54,55,56,57]. An extraneurite, such as the cell body, is analyzed using the DTI model. Intraneurites, including axons and dendrites, are analyzed using a stick model. Intracellular volume fraction (Ficvf) is a marker of neurite density, and orientation dispersion index (ODI) is a marker of neurite dispersion. DTI parameters, including FA and AD, depend on both brain neurite density and dispersion. FA reduction may occur due to decreased neurite density or increased neurite ODI, which can only be assessed via NODDI [53,58].

NODDI has been used to analyze various physiological and pathological conditions. In physiological situations, increased neurite ODI is associated with brain development, whereas reduced neurite density is linked with brain aging [53]. Structural alterations in neurites have been linked to numerous neurological disorders [59]. The value of the gray-matter ODI is reportedly significantly lower in the temporal pole, anterior parahippocampal gyrus, and hippocampus of patients with schizophrenia than in healthy controls [60]. Brain tumors with high Ficvf in the tumor parenchyma (icvfTP) and low Ficvf in the peritumoral region (icvfPT) are more likely to be high-grade gliomas, whereas lesions with low icvfTP and high icvfPT are more likely to be low-grade gliomas [61]. Decreased dendritic arborization, dendritic spine loss, and synapse loss have been observed in neuropathological studies of Alzheimer’s disease and are associated with cognitive deficits. The ODI values of multiple cortical regions were significantly lower in patients with Alzheimer’s disease [62]. The cortical ODI is associated with Mini-mental State Examination performance. In the injured brain in mouse models of closed traumatic brain injury, only the NODDI-derived Ficvf was significantly lower [63]. The ODI values computed using the NODDI model were nearly identical to those obtained via electron microscopy. Ficvf was related to cognitive functioning in patients with moyamoya disease, and all NODDI parameters were significantly correlated with positron emission computed tomographic parameters and clinical severity [64]. Neurite density and network complexity decreased with the increasing severity of the ischemic burden and increased interstitial fluid. ODI was higher in gadolinium-enhancing multiple sclerosis lesions than in nonenhancing lesions [65]. Some other studies have indicated that NODDI may be useful in determining seizure focus in patients with focal cortical dysplasia [66]. Decreased neurite density has been observed in temporal lobe epilepsy, mainly in the ipsilateral temporal areas [67].

However, to the best of our knowledge, no study has evaluated NODDI in patients with migraine. Therefore, we examined the DTI and NODDI in patients with migraine and compared them with those of healthy controls.

## 2. Materials and Methods

### 2.1. Study Population

Overall, 29 patients with migraine (mean age: 44.5 years; two men) and 23 healthy controls (mean age: 44.9 years; six men) were recruited (Table 1). All patients were diagnosed and treated at the Mito Medical Center, Mito Kyodo General Hospital, University of Tsukuba, Japan. The patients met the ICHD-3 criteria for the diagnosis of migraine [2]. The ICHD-3 was also used to diagnose EM, CM, and medication overuse headache (MOH). Migraine was episodic in 17 patients and chronic in 12 patients, and MOH was diagnosed in 4 migraine patients (Table 2 and Table 3). The mean ages of the EM and CM groups were 46.6 and 41.4 years, respectively, with the difference being not statistically significant. The mean ages of MOH and non-MOH patients were 37.8 and 45.6 years, respectively, with the difference being not statistically significant. The mean disease duration of all migraine patients was 21.4 years. The mean disease durations of EM and CM were 23.9 and 17.6 years, respectively, and this difference was not statistically significant. The mean disease durations of MOH and non-MOH patients were 7.5 and 23.6 years, respectively, with the difference being statistically significant (*t*-test, *p* < 0.01). There was no history of neurological disease, severe headache, head trauma, psychiatric disorder, or any intracranial abnormalities on anatomical MRI in the healthy controls. None of the migraine patients had any clinical and radiological neurological diseases such as stroke and dementia or any systematic diseases such as hypertension and malignancy. We obtained institutional review board approval prior to conducting this study (No. 20-56) on 2021-March-25 and informed consent from the patients.

### 2.2. Imaging Protocols

The patients and healthy controls underwent DTI and NODDI using a 3.0-Tesla MR scanner (Siemens, Erlangen, Germany) with a 32-channel head coil. All participants were instructed to close their eyes and not fall asleep. Structural imaging, including T1-weighted volume using magnetization-prepared rapid acquisition with gradient echo and T2 (fluid-attenuated inversion recovery), was performed. For T1-weighted imaging, the following parameters were used: repetition time (TR)/echo time (TE) = 2300/2.32 ms, 192 slices, and slice thickness = 0.9 mm. The following single-shot echo planar imaging parameters were used for diffusion-weighted acquisition: TR/TE = 7500/95 ms; matrix size = 128 × 128; 50 axial slices; slice thickness = 3 mm with no gap; field of view = 230 × 230 mm; 65 axial slices; number of excitations = 1; generalized autocalibrating partially parallel acquisitions (GRAPPA) factor = 2; 30 non-collinear axes; and b values of 0, 1000, and 2000 s/mm^2^. The FA, MD, AD, RD, Fiso, ODI, and Ficvf in the whole brain were analyzed using tract-based spatial statistics (TBSS). The region of interest (ROI) was placed on the corpus callosum. All MR images were examined during the interictal phase of the migraine.

### 2.3. Statistical Analysis

Statistical analyses were performed using SPSS version 28.00 (IBM, Tokyo, Japan). Fisher’s exact test and the two-sample unpaired t-test were used for the comparison of the sex and age distributions between the two groups. DTI and NODDI were analyzed using the randomize program of FMRIB, Oxford, UK Software Library (software 6.0v; http://www.fmrib.ox.ac.uk/fsl (accessed on 25 March 2021)). The significance level was set at a *p*-value of < 0.05. The mean FA, MD, AD, RD, Ficvf, and ODI in the ROIs were calculated and analyzed using the corresponding *t*-test program, MATLAB (R2017a, MathWorks, Natick, MA, USA).

## 3. Results

There were no significant differences in the DTI and NODDI parameters between the migraine patients and healthy controls. Additionally, there were no significant differences in the DTI and NODDI parameters between migraine patients with and without MOH. The Fiso was not significantly different between the EM and CM patients and between the MOH and migraine patients.

**Figure 1:**
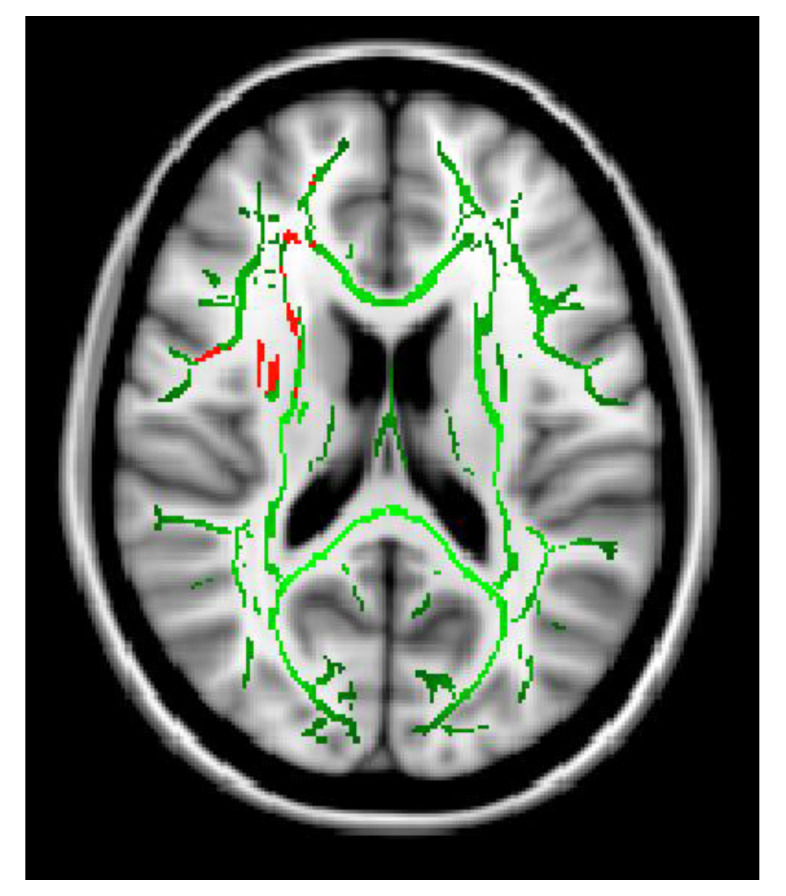
Ficvf (EM > CM): Green skeleton showed no difference, red skeleton showed difference at *p* = 0.07.

**Figure 2:**
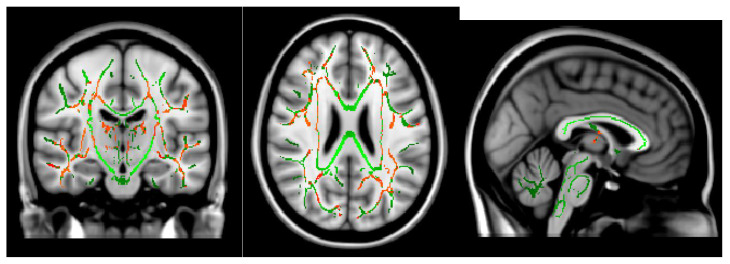
Ficvf (migraine > MOH): Green skeleton showed no difference, red skeleton showed difference at *p* < 0.2.

**Figure 3:**
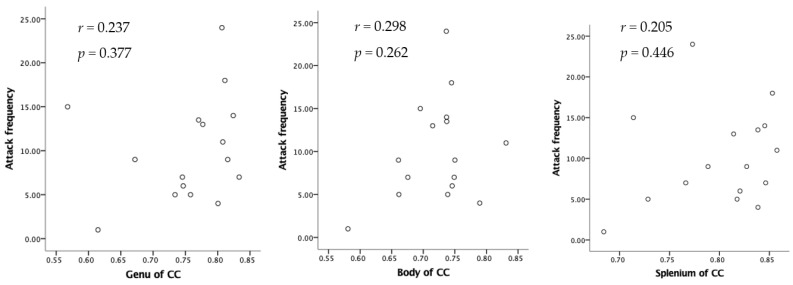
Relation between attack frequency and Ficvf in the corpus callosum.

The Ficvf of the CM patients was slightly lower than that of the EM patients in the right temporal lobe, although this difference was not statistically significant (Figure 1). The Ficvf of the patients with MOH was lower than that of patients with migraine in the bilateral cerebrum, although this difference was not statistically significant (Figure 2). No significant correlation was observed between migraine attack frequency and Ficvf in the genu, body, and splenium of the corpus callosum (Figure 3).

**Figure 4:**
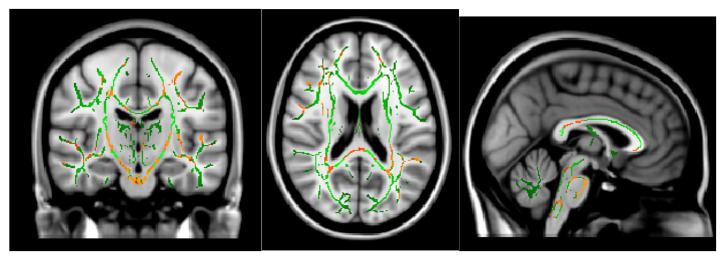
ODI (migraine > MOH) *p* < 0.05.

**Figure 5:**
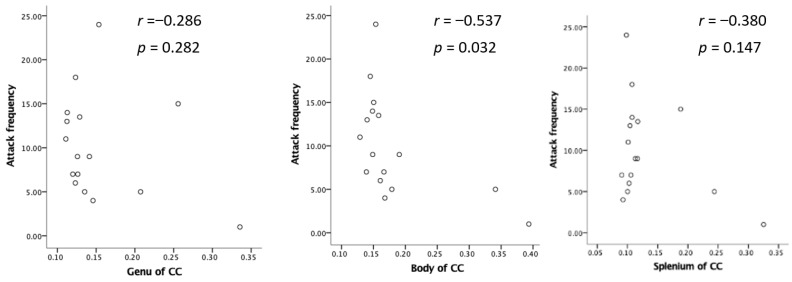
Negative relation between attack frequency and ODI.

The ODI in the bilateral cerebellum and brain stem was significantly lower in MOH patients than in patients with migraine (Figure 4). Migraine attack frequency and the ODI in the corpus callosum in the genu, body, and splenium showed negative correlations. In particular, the body in the corpus callosum showed a statistically significant correlation (Figure 5).

## 4. Discussion

In the present study, we did not find any significant differences in the DTI and NODDI parameters between migraine patients and healthy controls. Fiso has been reported as a marker of neuroinflammation [54,55,56,57]. In acute neural inflammatory diseases, including demyelinating diseases such as multiple sclerosis and neurodegenerative diseases such as Parkinson’s disease, neural inflammation precedes neural degeneration. Therefore, the fraction of free water volume increases before pathological microstructural changes are detectable with DTI. Given that we could not find any significant differences in Fiso, the neural tissue in patients with migraine does not demonstrate neural inflammation, or at least those that are detectable in NODDI. We did not observe the increase in free water volume in the neural tissue, which may have been caused by the fact that we only examined the migraine patients during the interictal period. During the migraine cycle, some parts of the nervous tissue, including the brainstem, hypothalamus, and trigeminal nerve, may demonstrate an increase in free water content.

We found that patients with MOH exhibited lower ODIs than those without MOH. Moreover, ODI in the corpus callosum was statistically negatively correlated with migraine attack frequency. These findings indicate that neurite dispersion is damaged in patients with MOH and CM.

MOH and CM showed pathological changes in DTI and fMRI using a 3.0-Tesla MRI scanner [52,68]. Schmitz et al. investigated the MRI findings of 28 adult female patients with migraine and compared them with those of age-matched female controls [69] using voxel-based morphometry for whole-brain analysis. Patients with migraine showed significantly reduced FA values in the superior frontal bole, middle frontal lobe, brain stem, and cerebellum as compared with the control patients. Individuals with long disease duration as compared with those with short have reduced frontal anisotropy. The differences between EM and CM groups have been demonstrated in various modalities, including electro- and magneto-physiological brain activity (M/EEG) and neurovascular and metabolic recordings from fMRI and positron emission tomography [70]. We also demonstrated significant differences between EM, CM, and MOH using DTI [47].

There are several methods to analyze the DTI data. The region of interest method is the most simple; the investigator can put the region of interest on the images. Only the selected area is analyzed, and the location and area are intentionally selected, so bias cannot be avoided. In the tract-specific analysis, specific tracts are selected and analyzed. Given that there are individual variations in brain morphology, the comparison between different individuals requires the standardization of brain morphology. Voxel-based analysis is a whole-brain analysis after standardization. Given that whole-brain analysis involves a large amount of data, a longer time for analysis is required. Some image transactions, including smoothing, are required. These image transactions and partial volume effects decrease the sensitivity of the differences. TBSS analysis is a new method for summarizing the whole-brain DTI data into white matter skeletons [71]. Whole-brain standardization was not required because only white matter skeletons were analyzed. In the present method, only limited data were analyzed within an acceptable time window. Moreover, this technique has low noise and high sensitivity. Therefore, the TBSS analysis is the standard method for analyzing DTI [71].

Szabo et al. performed a TBSS analysis of the MRI data of patients with migraine [72] and found reduced FA, increased MD, and increased RD in the right frontal white matter. They did not find any associations between migraine attack frequency or disease duration and the intensity of DTI structural changes.

Yu et al. also compared DTI findings of 20 migraine patients without aura with those of 20 age-, education-, and sex-matched healthy volunteers [73]. Migraine patients without aura showed significantly lower FA, MD, and AD in multiple brain regions, specifically in the genu, body, and splenium of the corpus callosum, than the healthy controls. Some of these white matter changes were significantly correlated with headache duration and frequency. Given that a difference in RD was observed, they speculated that these decreases in FA, AD, and MD may reveal axonal loss in migraine patients without aura.

Li et al. examined the DTI data of 12 migraine patients without a depressive/anxious disorder, 12 patients complicated with depressive/anxious disorder, and 12 age- and sex-matched healthy individuals [74]. The FA values from the migraine groups were significantly lower than those from the control group, and the FA values from the group complicated with a depressive/anxious disorder were significantly lower than those from the group without a depressive/anxious disorder. This study indicated common pathological changes in the neural microstructure among patients with migraine and depressive/anxious disorders.

Rocca et al. also analyzed the DTI data of seven migraine patients with aura and eight migraine patients without aura and healthy controls [75]. No difference was found for any white matter bundles between the healthy controls and migraine patients without aura. However, the migraine patients with aura had reduced FA of both optic radiations as compared with healthy controls and reduced average FA of the right optic radiation as compared with migraine patients without aura. These findings indicate that aura may cause neural damage in optic radiation.

Microstructural changes were observed within the migraine attack cycle. During the interictal phase, migraine patients without aura had shown significantly higher FA and slightly lower MD values in the bilateral thalami than healthy volunteers [76]. These abnormal values normalized during an attack. They speculated that these changes were related to plastic peri-ictal modifications in regional branching and the crossing of neural fibers. Given that FA is affected by various neural tissues, these FA changes could not be explained only by the neural changes, but rather, the glial changes may contribute to these results.

A previous study performed a voxel-based morphometry analysis of structural T1-weighted MRI over the course of the migraine cycle [77]. Interictally, migraine patients without aura had a significantly lower gray matter density within the right inferior parietal lobule, right temporal inferior gyrus, right superior temporal gyrus, and left temporal pole than healthy volunteers. Ictally, gray matter density increased with the left temporal pole, bilateral insula, and right lenticular nuclei. These rapid changes in gray matter density may not be explained by the neural changes in the axon or cell body; however, spinal change could be responsible.

Yuan et al. investigated DTI and resting-state functional connectivity at the same time in migraine patients without aura [78]. Twenty-one migraine patients without aura and 21 age-, sex-, and education-matched healthy controls were examined. The results of the TBSS analysis revealed reduced FA values in the genu and splenium of the corpus callosum in migraine patients. Functional connectivity analysis showed decreased interhemispheric functional connectivity of the anterior cingulate cortex in migraine patients without aura as compared to the healthy controls. The reduced FA values of the genus of the corpus callosum correlated with decreased interhemispheric resting-state functional connectivity of the anterior corpus callosum. They discussed how reduced FA of the corpus callosum modulates interhemispheric connectivity in migraine patients without aura.

Most previous DTI studies were cross-sectional; therefore, these studies could not demonstrate the longitudinal change. Liu et al. examined these structural changes between repeated observations that were 1 year apart in a group of early-stage migraine patients without aura [40]. A gray matter volume reduction was observed in the dorsolateral and medical regions of the superior frontal gyrus, orbitofrontal cortex, hippocampus, precuneus, and primary and secondary somatosensory cortices. No significant differences in the FA, AD, RD, and MD of white matter were observed in the migraine patients without aura within a year. They speculated that the gray matter volume reduction in sensory-discriminative brain regions may occur in migraine patients in its early stage and that the white matter evolves slowly during migraine chronicity. DTI mainly reflects white matter structures; therefore, DTI may not be able to detect the early changes in the sensory cortex in patients with migraine.

Most previous DTI studies used a 3.0-Tesla MRI scanner. We have reported significant DTI changes using 1.5-Tesla MRI for patients with MOH and CM [47]. This study revealed that even 1.5-Tesla MRI could demonstrate significant imaging differences between patients with migraine and healthy controls.

In the present study, DTI revealed no abnormalities between patients with migraine and healthy controls. This discrepancy from the results of a previous study may be due to the different patient populations and the number of patients analyzed. However, NODDI demonstrated significant changes in patients with MOH and CM. Therefore, NODDI may be more sensitive than DTI for investigating neurite changes in patients with migraine.

Some fMRI studies have revealed dynamic changes in functional connectivity during a migraine attack cycle [37]. Various neuroimaging studies including arterial spin labeling, positron emission tomography, and DTI reported significant changes in migraine’s pre-ictal or premonitory phase [79]. One DTI study demonstrated interictal diffusivity fluctuation in the midbrain, dorsomedial pons, and spinal trigeminal nucleus over the migraine cycle [80]. These dynamic changes seem not to be caused by neural or axonal anatomical changes. Contrarily, neurite change may occur during a migraine attack cycle, although this speculation should be examined in the future.

If all neurites are equally damaged during a migraine attack, both neurite density and dispersion should decrease. However, the Ficvf in the corpus callosum showed no statistically significant correlation with migraine attack frequency. These results indicate that a high migraine attack frequency caused considerable damage to neurite dispersion as compared with neurite density. This may be caused by neurite damage only in the cross-section with an axon. Therefore, our study results indicate the orientation preference of neurite damage during a migraine attack.

This study has several limitations. The study participants were recruited from a single institution. Consequently, the study population, especially for the patients with MOH, was small and limited to Japanese individuals. Moreover, most patients were adults with a long history of migraine disease and had been medically treated for it. However, our study demonstrated significant findings. In the future, we plan to perform NODDI analyses for various populations of patients with migraine. Furthermore, we did not perform imaging during migraine attacks. Finally, our cross-sectional study compared patients with migraine with healthy controls. Thus, we do not have any longitudinal data regarding therapeutic interventions. Future investigations on the dynamic and longitudinal changes in NODDI findings in patients with migraine are warranted. We did not have any pathological data. In the future, a pathological study to demonstrate neurite damage in patients with migraine is expected.

## 5. Conclusions

Our study demonstrated that patients with MOH exhibited lower ODIs than those without MOH. Neurite damage in patients with CM and MOH was not observed in the DTI study. Therefore, a high migraine attack frequency and MOH caused considerable damage to neurite dispersion as compared with the neurite density. This may be caused by neurite damage only in the cross-section with an axon. Therefore, our study results indicate the orientation preference of neurite damage in patients with migraine. NODDI may be useful in understanding the pathophysiology of migraine.

## Figures and Tables

**Table 1:** The characteristics of migraine patients and healthy volunteers.

	Migraine	Healthy Volunteer	*p*
n	29	23	-
Male/female	2/27	6/17	ns
Mean age ±SD	44.5 ± 13.5	44.9 ± 12.7	ns
Duration ± SD	21.4 ± 15.7	-	-
MOH(Yes/No)	4/25	-	-
EM/CM	17/12	-	-

**Table 2:** Characteristics of EM and CM patients.

	EM	CM	*p*
n	17	12	
Male/female	0/17	1/11	
Mean age +SD	46.6 ± 14.6	41.4 ± 11.0	0.3
Duration +SD	23.9 ± 15.4	17.6 ± 15.3	0.32

**Table 3:** Characteristics of MOH and non-MOH patients.

	MOH	Non-MOH	*p*
n	4	25	
Male/female	0/4	1/24	
Mean age + SD	37.8 ± 7.1	45.6 ± 13.9	0.155
Duration + SD	7.5 ± 4.6	23.6 ± 15.7	<0.01

## Data Availability

The data are not publicly available due to privacy or ethical restrictions.
